# Decrease in Serum Urate Level Is Associated With Loss of Visceral Fat in Male Gout Patients

**DOI:** 10.3389/fendo.2021.724822

**Published:** 2021-09-14

**Authors:** Zijing Ran, Xiaomei Xue, Lin Han, Robert Terkeltaub, Tony R. Merriman, Ting Zhao, Yuwei He, Can Wang, Xinde Li, Zhen Liu, Lingling Cui, Hailong Li, Aichang Ji, Shuhui Hu, Jie Lu, Changgui Li

**Affiliations:** ^1^Shandong Provincial Key Laboratory of Metabolic Diseases and Qingdao Key Laboratory of Gout, The Affiliated Hospital of Qingdao University, Qingdao, China; ^2^Department of Endocrinology and Metabolism, The Affiliated Hospital of Qingdao University, Qingdao, China; ^3^Institute of Metabolic Diseases, Qingdao University, Qingdao, China; ^4^San Diego VA Healthcare System, San Diego, CA, United States; ^5^Department of Medicine, University of California San Diego, La Jolla, CA, United States; ^6^Division of Clinical Immunology and Rheumatology, University of Alabama Birmingham, Birmingham, AL, United States; ^7^Department of Biochemistry, University of Otago, Dunedin, New Zealand; ^8^Department of Nutrition, The Affiliated Hospital of Qingdao University, Qingdao, China; ^9^Shandong Provincial Clinical Research Center for Immune Diseases and Gout, Qingdao, China

**Keywords:** visceral fat, gout, hyperuricemia, obesity, urate-lowering therapy

## Abstract

**Objective:**

To clarify the relationship between serum urate (SU) decrease and visceral fat area (VFA) reduction in patients with gout.

**Methods:**

We retrospectively analyzed 237 male gout patients who had two sets of body composition and metabolic measurements within 6 months. Subjects included had all been treated with urate-lowering therapy (ULT) (febuxostat 20–80 mg/day or benzbromarone 25–50 mg/day, validated by the medical record). All patients were from the specialty gout clinic of The Affiliated Hospital of Qingdao University. The multiple linear regression model evaluated the relationship between change in SU [ΔSU, (baseline SU) – (final visit SU)] and change in VFA [ΔVFA, (baseline VFA) – (final visit VFA)].

**Results:**

ULT resulted in a mean (standard deviation) decrease in SU level (464.22 ± 110.21 μmol/L at baseline, 360.93 ± 91.66 μmol/L at the final visit, *p <*0.001) accompanied by a decrease in median (interquartile range) VFA [97.30 (81.15–118.55) at baseline, 90.90 (75.85–110.05) at the final visit, p < 0.001]. By multiple regression model, ΔSU was identified to be a significant determinant variable of decrease in VFA (beta, 0.302; *p* = 0.001).

**Conclusions:**

The decrease in SU level is positively associated with reduced VFA. This finding provides a rationale for clinical trials to affirm whether ULT promotes loss of visceral fat in patients with gout.

## Introduction

Gout is a global public health challenge (1) whose rise in prevalence has paralleled population trends for overweight and obesity (2). Elevated serum urate (SU) [hyperuricemia (HU)] is believed to be a substantial contributor to visceral fat accumulation (3). In most mammals, SU levels are maintained at 60–180 μmol/L due to the presence of uricase (4). However, in the Middle Miocene, silencing mutations of the *uricase* gene in human ancestors led to elevated SU (5, 6). Loss of uricase might have provided a survival advantage by amplifying the effects of fructose to enhance fat stores partly because of the ability of fructose to generate urate during metabolism (7). As hypothesized by Neel (8), *uricase* may represent the first example of a “thrifty gene,” the loss of which may explain the current epidemic of obesity by increasing our susceptibility to fat accumulation (9).

Data from 2007 to 2008 National Health and Nutrition Examination Survey (NHANES-III) suggest an elevated proportion with obesity [body mass index (BMI) ≥30] among individuals with gout compared to non-gout individuals (53% vs. 32.8%) (10). Patients with gout from NHANES-III had three times odds ratio of metabolic syndrome (11). Conversely, obesity is an important risk for incident gout (12). Moreover, accumulation of visceral adipose tissue is associated with deterioration of metabolic health (13) and the development of cardiovascular disease (14). From a health check-up examination study, it was shown that visceral fat obesity (defined as visceral fat area, VFA >100 cm^2^), measured using bioelectrical impedance analysis, was observed more frequently in patients with gout compared with healthy controls (15). A cross-sectional study of 867 patients with type 2 diabetes showed HU to be positively correlated with central body fat distribution, especially visceral adipose tissue (r = 0.328, *p* < 0.001) (16).

Given the high prevalence of visceral obesity in gout patients, understanding the effects of change in serum urate level on body composition is of critical importance. Recently, a longitudinal research study reported a modest association between change in SU and change in BMI (r = 0.22, *p* = 0.003) and change in waist circumference (r = 0.17, *p* = 0.05), even though the change in SU was relatively small (281 ± 69 at baseline vs. 262 ± 58 μmol/L at 1-year postpartum, p = 0.01) (17). Thus far, no clinical evidence shows that ULT can improve visceral adiposity, despite experimental data suggesting that elevated SU levels facilitate fat accumulation in mice (7). Therefore, the purpose of current retrospective study was to determine the relationship between decreased serum urate level and loss of visceral fat in patients with gout.

## Patients and Methods

### Study Design and Population

This is a clinical delivery population-based retrospective study. Patients from the dedicated gout clinic of The Affiliated Hospital of Qingdao University from August 2017 to August 2019 were retrospectively studied from the Biobank Information Management System (BIMS, Haier, China) clinical electronic medical record system. Every patient voluntarily provided their written informed consent to import their electric health records into the BIMS, further used in scientific research.

Data were extracted for patients who met the following criteria: (a) received ≥ 2 body composition measurements within 6 months; (b) age ≥ 18 years; and (c) male, to minimize the influence of gender confounding as the sex differences in fat distribution (18). Patients were excluded if they were administrated with orlistat (antiobesity drug). All patients met the American College of Rheumatology (ACR)/European League Against Rheumatism (EULAR) clinical classification criteria for gout (19) and accepting urate-lowering treatment during the observational time. The study was approved by the research ethics committee of the Affiliated Hospital of Qingdao University (#QYFYWZLL 26298).

### Measurements

Clinical characteristics included age, systolic and diastolic blood pressure (BP), comorbid disease, and concomitant medications. Fasting blood samples were collected from 7:00 to 9:00 a.m. Serum samples were analyzed on an automatic biochemical analyzer (TBA-40FR, Toshiba Company, Japan). Routine laboratory measurements included SU, alanine aminotransferase, aspartate aminotransferase, glucose, triglyceride, total cholesterol, creatinine, and blood urea nitrogen. BP was measured on the arm with an automatic sphygmomanometer while the participant was seated (Omron Colin).

We extracted body composition data, including body fat (BF), percentage of body fat (PBF), VFA, and skeletal muscle mass (SMM), from an InBody S10 body composition analyzer (InBody Co., Seoul, South Korea), a BIA device that is portable, non-invasive, and non-radiation. The effectiveness of bioelectrical impedance analysis in body composition measurement agrees well with the gold standard method—computed tomography (CT) (20). Included subjects’ body composition measurements were all measured on the day of blood sample collection.

BMI was calculated as the weight divided by the square of the height (kg/m^2^), and ≥24 kg/m^2^ was defined as overweight and ≥28 kg/m^2^ as obesity, according to Chinese criteria (21, 22). eGFR was calculated as follows: male, Cr ≤ 0.9 mg/dl, eGFR = 141 × (Cr/0.9)^−0.411^ × (0.993)^age^; Cr > 0.9 mg/dl, eGFR = 141 × (Cr/0.9) ^−1.209^ × (0.993)^age^ (23). Chronic kidney disease (CKD) was defined by a reduced eGFR to <60 ml/min/1.73 m^2^ as suggested by Kidney Disease Outcomes Quality Initiative (K/DOQI) clinical practice guidelines (24). Hyperlipidemia, diabetes, and hypertension were defined as per definitions at the time of cohort entry or being prescribed specific medications for each of them. Fatty liver was diagnosed by abdominal ultrasound or reported in the medical history.

### Statistical Analysis

All analyses were performed using SPSS version 26.0 (IBM, Armonk, NY, USA). Two-tailed probability values <0.05 were considered statistically significant. Before statistical analysis, continuous variables were tested for a normal distribution using the Kolmogorov–Smirnov test or Shapiro–Wilk test. Data were presented as mean ± standard deviation (SD), median (interquartile range, IQR), or number (percentage). Changes (Δ) were calculated as the (baseline level) – (final visit level). Comparisons of variables between baseline level and final visit level were undertaken using paired-sample *t-*tests or Wilcoxon rank tests as appropriate. To observe the relationship between ΔSU and ΔVFA, participants were divided according to quartiles of ΔSU (Q1, <25 μmol/L; Q2, 25–109 μmol/L; Q3, 109–189 μmol/L; and Q4, >189 μmol/L). Changes in visceral fat area, body fat mass, waist circumference, and body mass index were analyzed for each quartile of ΔSU.

Simple linear regression analyses were conducted to evaluate the respective associations of changes in metabolic parameters between visits, baseline characteristics, medical history, and concomitant medication uses, with change in visceral fat area between visits. Multiple linear regression analysis was performed to examine relations among variables by using ΔVFA as a dependent variable to evaluate the role of the parameters in VFA loss. Variables with *p* < 0.20 were selected in multiple linear regression analysis. To control for the potential confounding effects of concomitant medication, antihypertensive, lipid, glucose-lowering, and liver–kidney-protecting medication use were adjusted in Model 2. Tophus, fatty liver, chronic vascular disease, and medication use were included in regression analysis as dichotomous variables, and others were continuous variables. The dichotomous variables were coded 1 for the presence and 0 for the absence of the factor.

## Results

### Basic Characteristics

**Figure 1:**
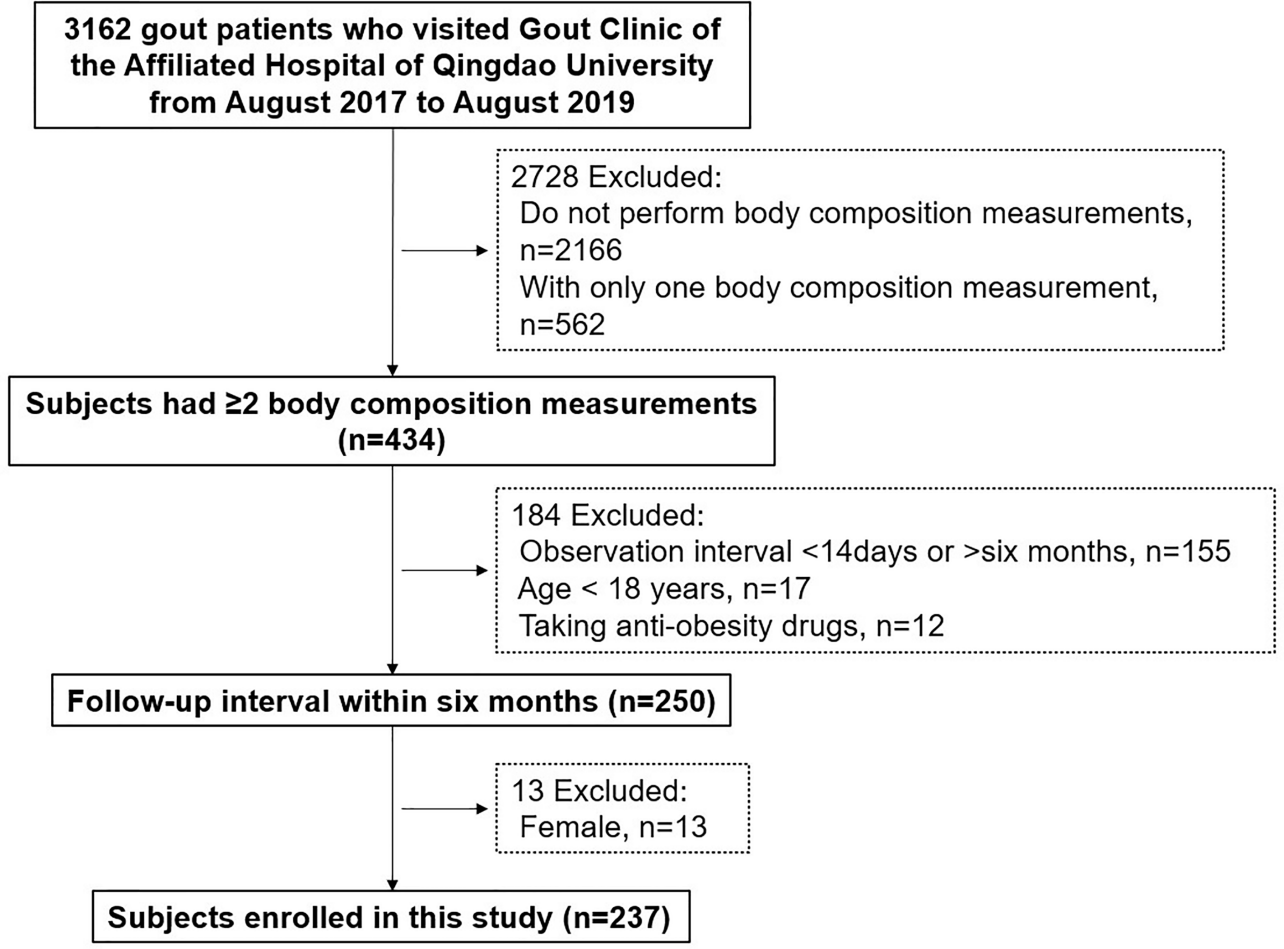
Study flow chart.

A total of 3,162 gout patients were screened. Patients were excluded because of the following criteria: individuals without (n = 2,166) or only a single body composition measurement (n = 562), taking antiobesity drugs (n = 12), age <18 years (n = 17), follow-up interval <2 weeks or >6 months (n = 155) and female (n = 13). After exclusion, 237 individuals were considered eligible for this study (**Figure 1**).

Anthropometric and metabolic characteristics of the study patients at baseline and final visit levels are presented in **Table 1**. The mean age of patients was 43.4 years, and the median (IQR) observation interval was 12 (9.0–14.3) weeks. Patients who were overweight (BMI ≥24) and obese (BMI ≥28) were 190 (80.2%) and 76 (32.1%), respectively. Eighty-one (34.2%) patients had hypertension, 12 (5.1%) with diabetes, 127 (53.6%) with hypertriglyceridemia, 41 (17.3%) with fatty liver, 6 (2.5%) with cardiovascular diseases, and 22 (9.3%) had tophus. Seven (3.0%) showed their eGFR <60 ml/min/1.73 m^2^.

**Table 1:** Main clinical characteristics of study patients at baseline and post ULT.

Characteristics	Baseline (n=237)	Final visit (n=237)	*P* value
***Patient demographics***			
Age, y	42.00 (33.50 ~ 53.50)	42.00 (33.50 ~ 54.00)	0.206
BMI, kg/m^2^	26.73 (24.84 ~ 28.72)	26.37 (24.51 ~ 28.40)	**<0.001**
Weight, kg	82.00 (75.00 ~ 89.35)	80.00 (74.00 ~ 87.5)	**<0.001**
Waist circumference, cm	94.50 (88.00 ~ 99.15)	93.10 (87.30 ~ 98.70)	**<0.001**
Visceral fat area, cm^2^	97.30 (81.15 ~ 118.55)	90.90 (75.85 ~ 110.05)	**<0.001**
Body Fat, kg	22.70 (19.10 ~ 26.95)	21.40 (17.60 ~ 25.05)	**<0.001**
Percent Body Fat, %	27.60 (24.60 ~ 31.55)	26.50 (23.30 ~ 29.75)	**<0.001**
Skeletal muscle mass, kg	32.94 ± 4.42	33.35 ± 4.25	**<0.001**
Systolic BP, mm Hg	132.40 ± 16.28	130.81 ± 13.80	0.083
Diastolic BP, mm Hg	83.63 ± 10.62	81.84 ± 10.72	**0.004**
Serum urate, μmol/L	464.22 ± 110.21	360.93 ± 91.66	**<0.001**
ALT, U/L	28.00 (20.00 ~ 44.00)	24.00 (17.00 ~ 36.00)	**<0.001**
AST, U/L	21.00 (18.00 ~ 27.00)	21.00 (17.00 ~ 25.50)	**0.003**
Glucose, mmol/L	5.43 (5.13 ~ 5.75)	5.36 (5.05 ~ 5.68)	0.054
Triglyceride, mmol/L	1.80 (1.21 ~ 2.77)	1.71 (1.14 ~ 2.49)	**0.001**
Total cholesterol, mmol/L	4.87 ± 0.96	4.75 ± 0.90	**0.015**
Blood urea nitrogen, mmol/L	4.50 (3.60 ~ 5.50)	4.80 (4.00 ~ 5.60)	**0.004**
Creatinine, μmol/L	81.00 (74.50 ~ 92.00)	82.00 (74.00 ~ 91.00)	0.977
eGFR, mL/min/1.73 m^2^	97.32 ± 17.51	97.27 ± 17.53	0.939
Patients achieving the SU target (<360 μmol/L), n (%)	49 (20.7%)	142 (59.9%)	**<0.001**
***Medical history***			
Tophus, n (%)	22 (9.3%)		
Overweight (BMI ≥24), n (%)	190 (80.2%)		
Obesity (BMI ≥28), n (%)	76 (30.1%)		
Chronic kidney disease, n (%)	7 (3.0%)		
Hypertension, n (%)	81 (34.2%)		
Diabetes, n (%)	12 (5.1%)		
Nephrolithiasis, n (%)	16 (6.8%)		
Hypertriglyceridemia, n (%)	127 (53.6%)		
Cardiovascular disease, n (%)	6 (2.5%)		
Fatty liver, n (%)	41 (17.3%)		
***Concomitant medications***			
Benzbromarone, n (%)	151 (63.7%)		
Febuxostat, n (%)	86 (36.3%)		
Colchicine, n (%)	121 (51.1%)		
Anti-hypertensive drugs, n (%)	32 (13.5%)		
Glucose-lowering drugs, n (%)	6 (2.5%)		
Fenofibrate, n (%)	37 (15.6%)		
Atorvastatin, n (%)	19 (8.0%)		
Liver-protecting drugs, n (%)	32 (13.5%)		
Kidney-protecting drugs, n (%)	9 (3.8%)		

The values are presented as Mean ± SD, Median (IQR 25th-75th), or number (percentage) as appropriate. Bold indicates P < 0.05.

BMI, body mass index; BP, blood pressure; ALT, aspartate aminotransferase; AST, aspartate aminotransferase; BUN, blood urea nitrogen; eGFR, estimated glomerular filtration rate; ULT, Urate-lowering therapy.

During the study period, dosages for urate-lowering drugs were adjusted according to patients’ SU levels, kidney, and liver functions. Eighty (33.7%) of the patients had already taken urate-lowering agents at the time of the baseline body composition evaluation. Of all, 86 were treated with febuxostat (range from 20 to 80 mg/day), and 151 were treated with benzbromarone (range from 25 to 50 mg/day). The SU levels were decreased from 464.22 (110.21) μmol/L at baseline to 360.93 (91.66) μmol/L at the final visit. Accordingly, the proportion of patients achieving the SU target (<360 μmol/L) was increased from 20.7% to 59.9% (**Table 1**). More than half of these patients took colchicine to prevent gout flare (0.5 mg/day, n = 84 or 1.0 mg/day, n = 37). Thirty-seven (15.6%) were treated with fenofibrate, 19 (6%) with atorvastatin calcium, 32 (13.5%) with antihypertensive drugs, 6 (2.5%) with glucose-lowering drugs, and 9 (3.8%) with kidney-protecting drugs. Thirty-two (13.5%) were prescribed liver-protecting drugs due to the reported hepatoxicity of febuxostat.

### Comparisons of Characteristics After Urate-Lowering Treatment

**Figure 2:**
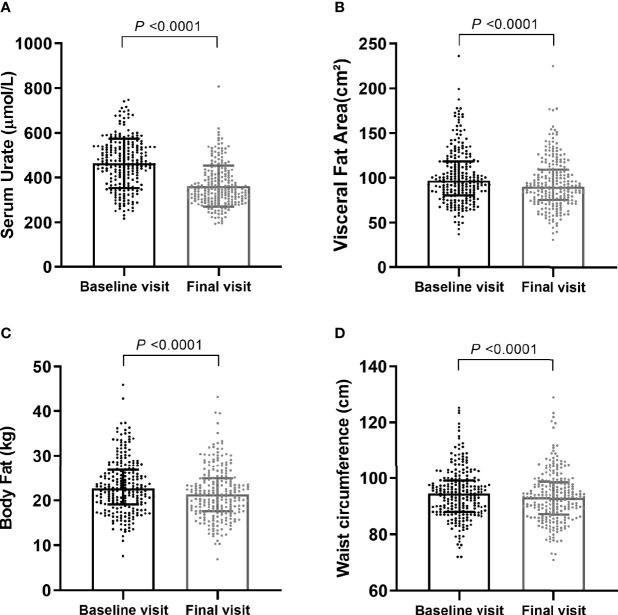
Comparisons of main characteristics after urate-lowering. Serum urate levels **(A)** were shown as Mean ± SD, visceral fat area **(B)**, body fat **(C)**, and waist circumference **(D)** were shown as Median (IQR 25th-75th). *P* values were calculated by paired-sample t-tests or Wilcoxon Rank tests as appropriate.

During the median 12 weeks follow-up, the mean (SD) SU dropped from 464.22 (110.21) μmol/L to 360.93 (91.66) μmol/L (*p* < 0.001) (**Figure 2A**). The median (IQR) of VFA also decreased, from 97.30 (81.15, 118.55) to 90.90 (75.86, 110.05) cm^2^ (*p* < 0.001) **(****Figure 2B****)**. Significant decreases were also detected in other body composition parameters, including body fat (*p* < 0.001) **(****Figure 2C****)**, WC (*p* < 0.001) (**Figure 2D**), BMI (*p* < 0.001), and lipid profiles—triglyceride (*p* = 0.001) and cholesterol (*p* = 0.003). Although statistically significant differences were shown in hepatic and other renal-related parameters at baseline and final visit, the average levels of these indicators were within the normal range throughout ULT (**Table 1**). None of the serum glucose, serum creatinine, eGFR, and systolic blood pressure (SBP) were significantly different (**Table 1**). In addition, 290 subjects (twice or more visits within 6 months with recorded body weight and BMI) showed same pattern of decreases in serum urate, body weight, and BMI (**Supplementary Methods**; **Supplementary Figure 1**).

### Comparisons of Characteristics Stratified by ΔSU Quartiles

**Figure 3:**
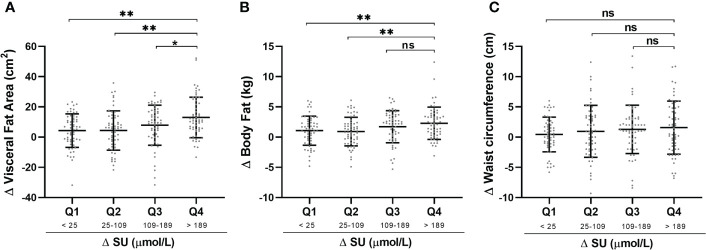
Comparisons of characteristics with ΔSU quartile stratification. Changes (Δ) in Visceral fat area **(A)**, body fat **(B)** and waist circumference **(C)** was calculated by baseline level-after treatment level and presented with Mean ± SD. Changes in SU was divided into quartiles with the following values: Q1: <25 μmol/L; Q2: 25-109 μmol/L; Q3: 109-189 μmol/L; Q4: >189 μmol/L. *P < 0.05, **P < 0.01, ns., no significance. SU, serum urate.

Patients were divided according to quartiles of ΔSU to assess the relations between ΔSU and body composition parameters (**Figure 3**). There were increasing trends toward greater loss in visceral fat area (**Figure 3A**), body fat (**Figure 3B**), and waist circumference (**Figure 3C**) with increasing quartiles of ΔSU. Q4 (>189 μmol/L) had a statistically significant decrease in VFA when compared to Q1 (<25 μmol/L), Q2 (25–109 μmol/L), and Q3 (109–189 μmol/L). We also assess the effects of xanthine oxidase inhibitor febuxostat and uricosuric agent benzbromarone on body composition. Neither VFA nor parameters including weight, WC, body fat, and skeletal muscle mass showed significant differences between these two urate-lowering agents (**Supplementary Table 1**).

### Correlation and Multiple Regression Analysis

In simple linear regression analysis, changes in serum urate, glucose, triglyceride, AST and baseline serum urate, and baseline visceral fat area were significant predictors of changes in visceral fat area (*p* < 0.05; **Table 2**). No relation was found between the type of ULT agents and ΔVFA. In multiple linear regression analysis, ΔSU and baseline VFA were identified to be a significant determinant variable of ΔVFA (Model 1: ΔSU, beta = 0.313, *p* < 0.001; VFA, beta = 0.380, *p* < 0.001; Model 2: ΔSU, beta = 0.302, *p* = 0.001; VFA, beta = 0.417, *p* < 0.001) **(****Table 2****)**.

**Table 2:** Simple and multiple linear regression of determinants of ΔVFA.

	Univariate analysis		Multivariate analysis			
			Model 1		Model 2	
	*Beta*	*P* ^†^	*Beta*	*P* ^††^	*Beta*	*P* ^††^
***Changes after ULT***						
ΔSerum urate, mg/dL	**0.314**	**<0.001**	**0.313**	**<0.001**	**0.302**	**0.001**
ΔGlucose, mmol/L	**0.154**	**0.021**	0.067	0.263	0.053	0.377
ΔTriglyceride, mmol/L	**0.202**	**0.002**	**0.125**	**0.045**	0.113	0.075
ΔTotal cholesterol, mmol/L	0.107	0.110	-0.018	0.777	-0.005	0.944
ΔALT, U/L	-0.029	0.667	–		–	
ΔAST, U/L	**-0.143**	**0.032**	-0.103	0.080	-0.114	0.058
ΔeGFR, mL/min/1.73m^2^	-0.091	0.172	-0.071	0.218	-0.064	0.331
ΔSystolic BP, mmHg	**0.150**	**0.025**	-0.074	0.247	0.084	0.246
ΔDiastolic BP, mmHg	0.094	0.159	0.070	0.327	0.005	0.940
***Baseline characteristics***						
Age, year	**-0.125**	**0.061**	-0.038	0.644	-0.029	0.743
Visceral fat area, cm^2^	**0.433**	**<0.001**	**0.380**	**<0.001**	**0.417**	**<0.001**
Serum urate, mg/dL	**0.206**	**0.002**	-0.136	0.120	-0.168	0.070
Glucose, mmol/L	-0.011	0.872	–		–	
Triglyceride, mmol/L	0.058	0.385	–		–	
Total cholesterol, mmol/L	0.045	0.505	–		–	
ALT, U/L	0.058	0.389	–		–	
AST, U/L	-0.080	0.235	–		–	
eGFR, mL/min/1.73m^2^	0.126	0.060	0.045	0.583	0.013	0.892
Systolic BP, mmHg	0.008	0.900	–		–	
Diastolic BP, mmHg	-0.092	0.218	–		–	
***Medical history***						
Duration of gout, year	-0.109	0.103	-0.043	0.505	-0.020	0.758
Tophus	-0.079	0.225	–		–	
Fatty Liver	0.042	0.528	–		–	
CVD	-0.029	0.668	–		–	
***ULT agents***						
Febuxostat	0.065	0.328	–		–	
Benzbromarone	-0.069	0.304	–		–	

Changes in VFA and metabolic parameters (Δ) was calculated by [baseline level] - [final visit level].

The data was analysed by simple linear regression and multiple linear regression. P^†^ values were calculated by simple linear regression and P^††^ values were calculated by multiple linear regression analysis. Bold indicates P < 0.05.

Model 1, Variables with P values < 0.20 in univariate analysis were selected in multivariable analysis.

Model 2, Adjusted for Model 1 plus use of febuxostat, benzbromarone, colchicine, anti-hypertensive drugs, lipid and glucose-lowering drugs, liver and kidney protecting drugs.

VFA, visceral fat area; ALT, aspartate aminotransferase; AST, aspartate aminotransferase; eGFR, estimated glomerular filtration rate; ULT, urate-lowering therapy. CVD, cardiovascular disease.

## Discussion

This retrospective longitudinal study provides the first clinical evidence to suggest that directly lowering SU by pharmacological ULT is associated with decreased visceral adiposity, focusing on patients with gout. Past studies reported the intercorrelation between hyperuricemia and obesity. Specifically, hyperuricemia was associated with visceral fat accumulation, and reduction in visceral fat was associated with reduction in SU (25). In subjects with overweight and obesity, hyperuricemia has been frequently observed (26–28). Moreover, hyperuricemia has been reported to have a predictive ability for weight gain (29). In a Japanese 5-year cohort, elevated SU levels were an independent predictor of developing obesity in people with hyperuricemia without any other comorbidity (30). More recently, Han et al. (31) presented a cross-lagged path analysis in the Chinese population and showed that the influence of SU on BMI was more significant than BMI on SU levels. Visceral adiposity, not subcutaneous fat, was associated with HU (32, 33).

In the present study, there were increasing trends toward greater loss in visceral fat area with increasing quartiles of ΔSU during the median 12 weeks follow-up. Q4 (>189 μmol/L) had a statistically significant decrease in VFA when compared to Q1 (<25 μmol/L), Q2 (25–109 μmol/L), and Q3 (109–189 μmol/L). A positive statistical association between visceral fat variance and serum urate decrease also was observed after adjustment for potential confounding variables and concomitant medication use (beta, 0.302; *p* = 0.001). As our previous study showed, SU levels were reduced by ~160 μmol/L in gout patients taking low-dose febuxostat (20 mg/day) or benzbromarone (25 mg/day) within 12 weeks (34). The ΔSU of Q4 in this study is a clinically relevant decrease and realizable in clinical practice. Long-term ULT is an effective treatment for gout patients, with 60%–80% of patients achieving the target SU level (<360 μmol/L) after 3 months of standardized ULT (35).

Functional effects of hyperuricemia on adipocytes have been suggested by prior mechanistic studies. The urate-anion exchanger transporter 1 (URAT1), which plays a key role in urate homeostasis in the proximal tubule (36), is also expressed in hepatocytes and adipocytes (37, 38) and transports urate into these cells. In hepatocytes, urate promotes the conversion of citrate to acetyl-CoA for *de novo* lipogenesis (7), buffering an increase in circulating free fatty acids. On the other hand, elevated intracellular SU levels directly and dose dependently induce excessive triglyceride accumulation in adipocytes through upregulating the expression of lipogenesis-related proteins and downregulating expression of lipolysis-related proteins (39). Both newly synthesized and existing free fatty acids in adipocytes convert into triglyceride and eventually deteriorate visceral obesity. An *in vivo* study has indicated that administration of the non-xanthine oxidoreductase (XOR) inhibitor, ULT uricosuric drug benzbromarone, can reverse this effect by reducing lipogenesis in adipocytes and decreasing triglyceride content (39). In addition, XOR is held to be involved in adipogenesis and contribute to metabolic syndrome (40). In this clinical study, our data showed no relation between the type of ULT agents and ΔVFA. There was no discernible difference in percent change in VFA between febuxostat (XOR inhibitor) and benzbromarone (uricosuric agent) treated groups. These results suggest that it is the decrease in SU by itself that mediates change in visceral adiposity.

Among patients who underwent bariatric surgery, preferential visceral fat mobilization only presented in those with excessive amounts of visceral adipose tissue before surgery (41). The energy-balance dynamics calculated based on a population-averaged model showed that adults with greater adiposity lost more weight with the same levels of change in energy intake (42). Our data show the same pattern. Baseline visceral fat level was a significant determinant variable for loss in VFA (beta = 0.417; *p* < 0.001), which indicated that gout patients with more visceral fat could lose more fat. Interestingly, a slight improvement in skeletal muscle mass was observed (32.94 ± 4.42 kg at baseline, 33.35 ± 4.25 at the final visit, *p <*0.001). Elevated insulin resistance was observed to be associated with greater loss of lean body mass (43). In addition, both clinical and experimental improvements in insulin resistance are aligned with decrease in SU level (44–46). These associations lead us to speculate that improved insulin resistance might play a role in skeletal muscle mass gain in gout patients undergoing ULT.

Weight loss is commonly recommended for patients with gout and hyperuricemia, and clinical evidence proved the effectiveness of this intervention (47, 48). However, bariatric surgery is only appropriate for severe obesity (BMI >40 kg/m^2^ or BMI of 35–40 kg/m^2^ with associated comorbid conditions). Moreover, adherence to diet-based weight loss strategy is usually low and difficult to sustain owing to the difficulty adapting to the physiological and neurohormonal aspects of weight loss (49). Our data suggest that decrease in SU level is a significant determinant variable of improvement of visceral obesity, which could be a potential benefit of ULT. This may be clinically useful in encouraging gout patients to accept urate-lowering therapy.

The primary strength of our study is that it was the first study to assess the correlation between changes in serum urate level and changes in visceral fat in Chinese male gout patients. Meanwhile, we acknowledge several limitations. First, this study was retrospective in nature, which did not allow us to reach a cause–effect relationship. Second, this single-center, single-ethnic study may not be generalizable to non-Chinese gout populations (including women). Third, dietary and physical information was not collected in our study. Participants seeking to start gout care may have changed their activity patterns and dietary intake, which would weaken the magnitude of association between changes in serum urates and changes in visceral fat. Currently, it is difficult for us to rule out the possibility of diet and exercise. Finally, we analyzed the data as a whole by ignoring ULT dosages and duration details. The effects of ULT duration on visceral fat improvement and the maximum reduction by lowering SU need to be evaluated prospectively.

In summary, our results indicate that decrease in serum urate level is associated with loss in visceral fat, especially in gout patients with an initially high baseline level of visceral fat. Our finding provides new insight into the improvement of abdominal obesity in patients with hyperuricemia and gout, which may provide additional emphasis to support initiation and adherence in ULT.

## Data Availability Statement

The raw data supporting the conclusions of this article will be made available by the authors, without undue reservation.

## Ethics Statement

The studies involving human participants were reviewed and approved by the research ethics committee of the Affiliated Hospital of Qingdao University (#QYFYWZLL 26298). The patients/participants provided their written informed consent to participate in this study.

## Author Contributions

CL and JL contributed to the study concept and design. CW, XX, and TZ contributed to the acquisition of data. ZR, JL, XX, LH, TM, RT, and CL contributed to drafting and modifying the manuscript. ZR, XL, LC, HL, AJ, and ZL interpreted the data. ZR, YH, JL, XX, and SH worked on the sample processing and the statistical analysis. CL has full access to all of the data in the study and takes responsibility for the integrity of the data and the accuracy of the data analysis. All authors contributed to the article and approved the submitted version.

## Funding

This work was sponsored by the National Natural Science Foundation of China (#81770869 and #31900413), National Key Research and Development Program (#2016YFC0903401), Shandong Province Key Research and Development Program (#2018CXGC1207), and Shandong Province Natural Science Foundation (#ZR2018ZC1053). Prof. Robert Terkeltaub was supported by NIH (#AR060772 and #AR075990) and the VA Research Service.

## Conflict of Interest

RT is funded by a research award from Astra-Zeneca and has consulted with Horizon, Selecta, SOBI, and Astra-Zeneca.

The remaining authors declare that the research was conducted in the absence of any commercial or financial relationships that could be construed as a potential conflict of interest.

## Publisher’s Note

All claims expressed in this article are solely those of the authors and do not necessarily represent those of their affiliated organizations, or those of the publisher, the editors and the reviewers. Any product that may be evaluated in this article, or claim that may be made by its manufacturer, is not guaranteed or endorsed by the publisher.

## Supplementary Material

Supplementary Figure 1 Comparisons of serum urate, weight, body mass index after urate-lowering treatment. Serum urate levels are shown as Mean ± SD, body weight and body mass index are shown as Median (IQR 25th-75th). *P* values are calculated by paired-sample t-tests or Wilcoxon Rank tests as appropriate.

## Abbreviations

BF, body fat; VFA, visceral fat area; WC, waist circumference; BMI, body mass index; BP, blood pressure; ALT, aspartate aminotransferase; AST, aspartate aminotransferase; BUN, blood urea nitrogen; eGFR, estimated glomerular filtration rate; CKD, chronic kidney disease; CVD, cardiovascular disease; ULT, urate-lowering therapy; SU, serum urate; HU, hyperuricemia; URAT1, urate-anion exchanger transporter 1; XOR, xanthine oxidoreductase; TG, triglyceride
