# Supplementary material for: Decrease in Serum Urate Level Is Associated With Loss of Visceral Fat in Male Gout Patients
Source: Front Endocrinol (Lausanne). 2021 Sep 14;12:724822. doi: 10.3389/fendo.2021.724822 (PMC8476917; doi:10.3389/fendo.2021.724822)
Supplement: Supplementary file 1 [file DataSheet_1.docx]

| **Supplementary materials**  **sMethods**  For the purpose of this study, we included only patients with two sets of body composition measurements. In order to verify the validity of our findings, we extract data from the excluded patients who met the following criteria: (a) age ≥18years; (b) males; (c) ≥ twice visit within six months with recorded body weight and BMI; (d) accepting ULT. Finally, 290 subjects were enrolled. We compared the serum urate levels, body weight and BMI in the 237 subjects as included group and 290 subjects as excluded group at baseline and final visits. Excluded group showed same pattern of decreases in serum urate, body weight and BMI.  **Supplementary Table 1.** **Percentage changes in serum urate and body compositions after ULT between febuxostat and benzbromarone.** | | | |
| --- | --- | --- | --- |
| **Percentage Changes** | **Febuxostat**  **(n=86)** | **Benzbromarone**  **(n=151)** | ***P*-value** |
| Δ% Serum urate, μmol/L | 18.55 (-5.71 ~ 39.55) | 25.24 (10.70 ~ 36.02) | 0.291 |
| Δ% Weight | 1.11 (-0.33 ~ 3.13) | 0.00 (-1.12 ~ 2.27) | 0.202 |
| Δ% Waist circumference, cm | 1.41 ± 4.43 | 0.91 ± 4.09 | 0.384 |
| Δ% Visceral fat area, cm^2^ | 7.42 (-1.85 ~ 14.74) | 8.61 (-0.71 ~ 15.14) | 0.548 |
| Δ% Body fat, kg | 5.31 (-1.95 ~ 12.95) | 7.69 (-1.18 ~ 13.47) | 0.677 |
| Δ% Skeletal muscle mass, kg | -1.26 (-3.76 ~ 1.66) | -1.64 (-3.54 ~ 0.83) | 0.352 |

(Δ) was calculated by baseline level-after treatment level. Δ% denotes percentage changes.

The values are presented as Mean ± SD or Median (IQR 25th-75th) as appropriate.

Abbreviations: ULT: Urate-lowering therapy
