# Supplementary figures and images for: Decrease in Serum Urate Level Is Associated With Loss of Visceral Fat in Male Gout Patients
Source: Front Endocrinol (Lausanne). 2021 Sep 14;12:724822. doi: 10.3389/fendo.2021.724822 (PMC8476917; doi:10.3389/fendo.2021.724822)

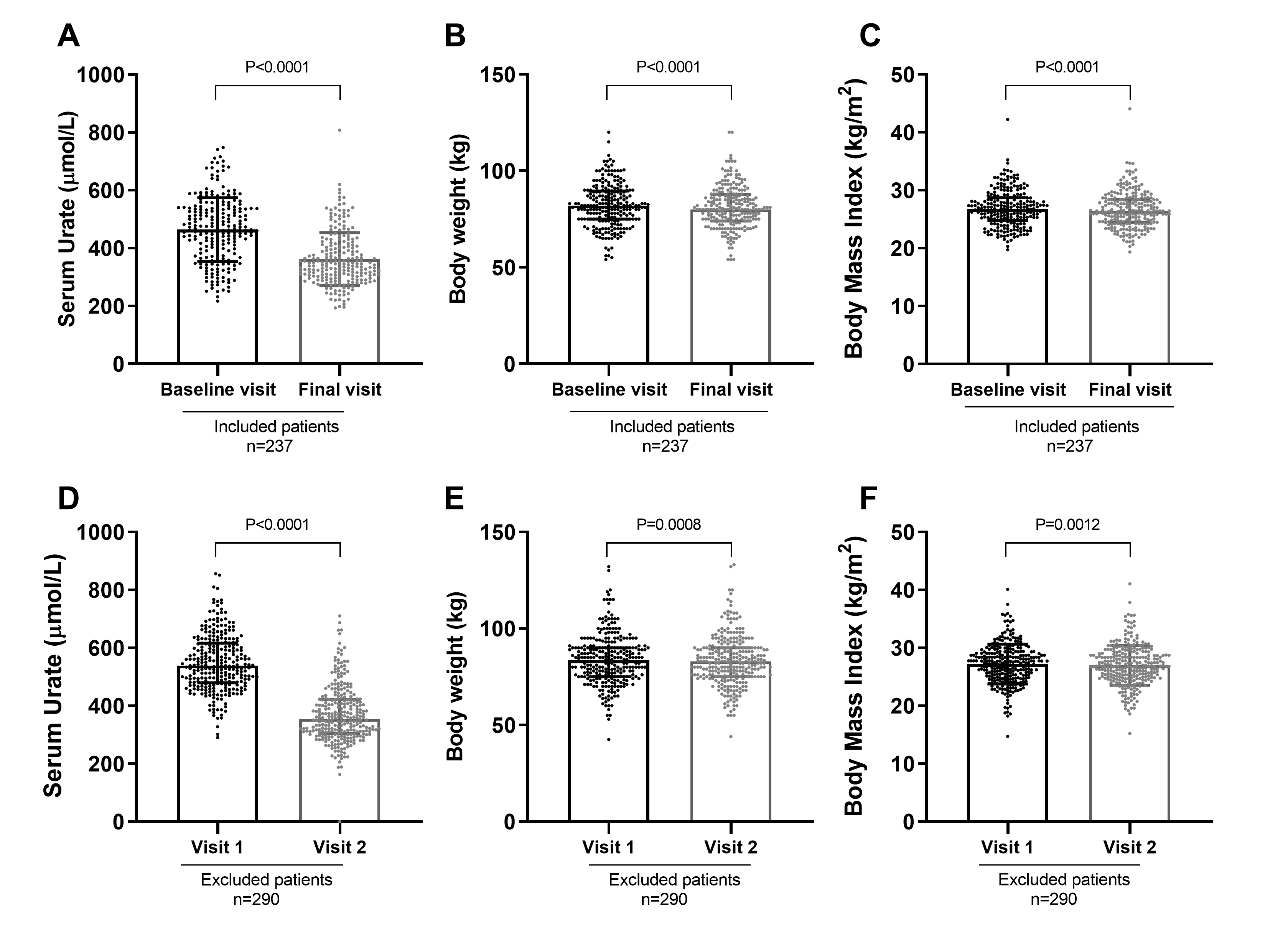

Supplement: Supplementary Figure 1 — Comparisons of serum urate, weight, body mass index after urate-lowering treatment. Serum urate levels are shown as Mean ± SD, body weight and body mass index are shown as Median (IQR 25th-75th). P values are calculated by paired-sample t-tests or Wilcoxon Rank tests as appropriate. [file Image_1.tif]
